# An Investigation on a Novel 3-RCU Flexible Micromanipulator

**DOI:** 10.3390/mi11040423

**Published:** 2020-04-17

**Authors:** Junnan Qian, Yangmin Li, Lukai Zhuge

**Affiliations:** 1 Tianjin Key Laboratory for Advanced Mechatronic System Design and Intelligent Control, Tianjin University of Technology, Tianjin 300384, China; junnanqian@163.com (J.Q.); zhugelk@foxmail.com (L.Z.); 2 Department of Industrial and Systems Engineering, The Hong Kong Polytechnic University, Hung Hom, Hong Kong 999077, China

**Keywords:** three revolute-cylindrical-universal (3-RCU), flexible hinge, flexible micro manipulation platform, lever amplification mechanism, finite element analysis, high precision

## Abstract

A novel type of spatial three revolute-cylindrical-universal (3-RCU) flexible micro manipulator is designed based on flexible hinges, and analyzed by finite element analysis (FEA). The piezoelectric actuators are adopted as driving devices in this platform, a new lever amplification mechanism is designed as its micro-displacement amplification mechanism, the workspace of the platform is enlarged, and the theoretical and simulation amplification ratios of the amplification mechanism are 3.056 and 2.985, respectively. The margin of error is just 2.3%. In space, the 3-RCU platform can realize the micro movement of three degrees of freedom. Also, the platform has a high carrying capacity, less motion loss, and the transmission efficiency is higher when the platform works. The decoupling performance, stress under extreme conditions and natural frequency of the platform are simulated by ANSYS Workbench software. A series of simulation analyses show the feasibility and security of the platform. The platform has good decoupling and working performance. The simulation results show that the platform has high simulation stiffness and high positioning accuracy.

## 1. Introduction

With the development of microelectronics, the miniaturization of devices, high integration of chips, and simplification of operations have become hot spots in micron(micro)/nanometer(nano) technology. The parallel micro operating platform, which combines micro-operation with parallel robot technology, has become a new direction of research. The micro operation platform has many advantages, such as good stability, high precision, high rigidity, and good bearing capacity [1,2]. The key part of micro-manipulator robot technology is to design a compact structure with large stroke, high precision, high flexibility, and easy miniaturization [3]. Generally, the precision positioning platform uses traditional rigid motion pairs, which are easily affected by the motion gap, and it is difficult to meet the requirements of high precision and structural reliability of the micro/nano platform [4]. The emergence of flexible hinges provides a solution to this problem and the systematic study of flexible hinges dates back to the late 1980s. A flexible mechanism is realized by a flexible hinge. The flexible mechanism is a new mechanism for transforming or transferring motion, force, and energy with a small deformation of material [5]. The flexible mechanism is easy to process, has no gap, friction, and wear, as well as high precision and better transmission efficiency. Thus, the flexible hinge is very suitable to replace the traditional rigid joints as the motion pair of the flexible micro-manipulator. Many flexible micro-operating platforms are driven by piezoelectric actuators (PZTs), which have broad application prospects in micro-manipulators. Piezoelectric actuators are simple in structure, easy to control, and high in resolution [6]. However, the output displacement of the piezoelectric actuators is too small, so it is necessary to add a micro-displacement amplifying mechanism. The amplifying mechanism should satisfy the large-stroke, high-precision design requirements of the micro-motion platform [7]. Because of the many advantages of parallel mechanisms [3,8,9], such as, they can meet the needs of micro-operating robots for high motion resolution, demonstrate fast response speed, have a small shape and high precision, based on previous researches [1,2,4,8,10,11,12,13,14,15,16,17], a new revolute-cylindrical-universal (3-RCU) parallel platform is designed. This is a feasible solution for the platform with high positioning accuracy. The platform can realize three-dimensional (3D) motion at micro/nano level and has the advantages of compact structure, high precision, and a good linear relationship.

## 2. The Overall Design of Flexible Micro-Operation Platform

### 2.1. Structural Design of the Platform

**Figure 1:**
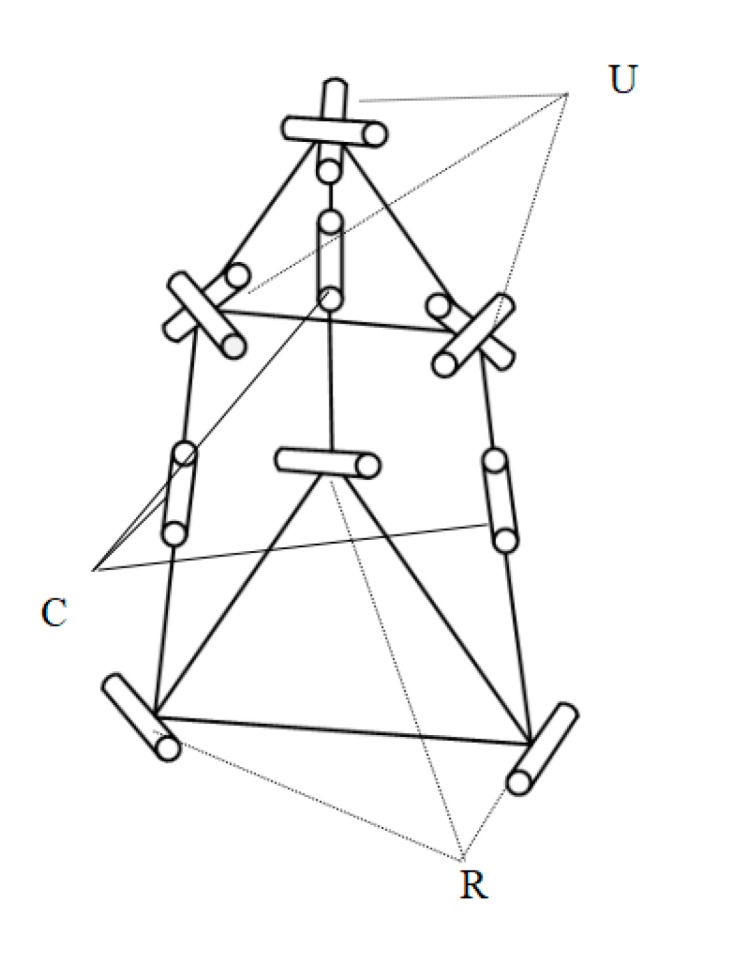
Structure diagram of micromanipulator platform.

A new three degrees of freedom (DOF) flexure parallel micromanipulation platform was designed. The micro platform consists of a moving platform, a static platform, and three identical branches. The three branched chains are distributed symmetrically with 120°. The platform with this distribution mode has good stability, and the motion decoupling of the mechanism is also good. The moving platform is connected with the hook hinge. In the middle is a new compound cylindrical pair, and the flexible rotation pair is connected with the static platform. During the movement of the platform, the closer the direction of the cylindrical pair moves to the vertical direction, the better the transmission effect of the flexible motion pair is. That is, the angle between the moving direction and the vertical direction of the branch chain is as small as possible. Overall, the optimal design method is to place the whole branch chain vertically, perpendicular to the two platforms. According to the arrangement position of branched-chain and flexible movement pairs on the branched-chain, the mechanism is named as 3-RCU flexible micro-operation platform. The mechanism diagram of the micro-manipulator platform is shown in Figure 1.

There are two initial designs for the platform configuration. Firstly, the amplifying mechanism is connected in parallel with the guiding mechanism to form a motion pair, and then placed at the end of the branch. Secondly, a flexible platform with a 3-PRS configuration is selected, and a composite bridge amplifying mechanism is applied as a micro-displacement amplifying mechanism of the platform. The three prismatic-universal-universal (3-PUU) [18] and three revolute-prismatic-spherical (3-RPS) [19] are the classical models of these two schemes, respectively. For the 3-PUU platform, the motion pair at the bottom, and the only part of the load can be transferred to the moving platform, which seriously affects the transmission efficiency of the platform. The flexible ball hinge used in the platform has very strict thickness requirements. If the thickness is too large, the range of rotation of the torsion angle will be affected. On the contrary, the bear stress of the platform under the extreme working environment is difficult to meet the requirements, and breakage may even occur. The platform designed is aimed at high-precision operation use in the fields of biological engineering medical surgery, optical fiber docking, etc. The platform with three degrees of freedom can realize rotation around two directions and movement along the z-axis. The most typical structure of the 2-rotate-1-translation (2R1T) type platform is the 3-RPS parallel mechanism [20,21]. In this paper, the parallel 3-RCU mechanism is adopted which is seldom used in the field of flexible precise positioning.

**Figure 2:**
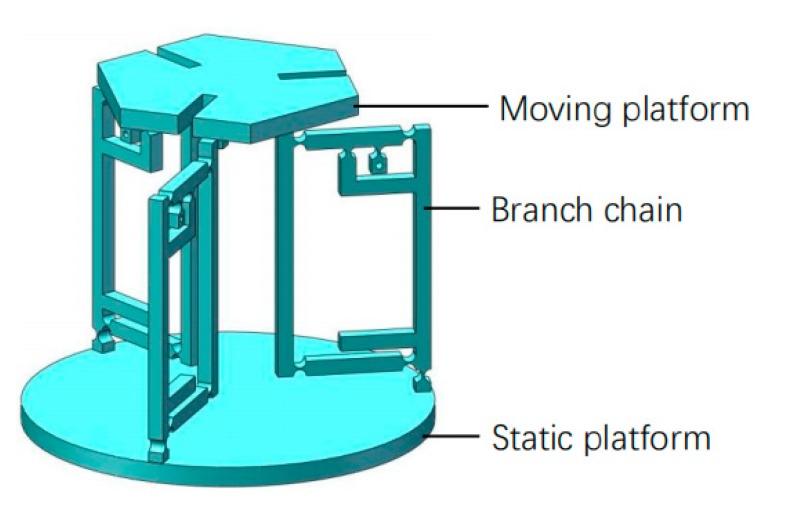
A three revolute-cylindrical-universal (3-RCU) flexible parallel micro operation platform.

The final model of the 3-RCU flexible parallel micro operation platform is shown in Figure 2. The compact flexible U-shaped hinge designed has great advantages in space, rigidity, and motion performance. Compared with the serial hooker hinge in the 3-PUU mechanism, the type of hooker hinge in this paper is more suitable for the miniaturization of structure design. Compared with the 3-RPS mechanism, the slotted design with three circular arrays can reduce the mass of the moving platform and increase the transmission performance of the end of the moving platform. The flexible cylindrical pair is used as an amplifier and a piezoelectric actuator is built into the amplifier mechanism, which not only reduces the size of the supporting chain and makes the structure compact, but also increases the stiffness of the mechanism and strengthens the bearing capacity of the supporting chain. By placing the branch chain vertically in space, the angle between the branch chain and the moving platform is reduced and the transmission effect of the platform will be better, which is conducive to improving the motion performance of the platform. In addition, compared with the composite bridge displacement amplification mechanism, the new type of lever amplifying mechanism designed in this paper contains fewer flexible hinges, so the platform has less motion loss, higher transmission efficiency, and better precision.

### 2.2. Calculation of Platform Degrees of Freedom

According to the mechanism diagram of the platform and the Kutzbach–Gubler [22] freedom degree calculation Equation (1), the degree of freedom of the platform is calculated:

(1) $$Μ=6\left(n-g-1\right)+\sum_{i=1}^{g}f_{i}+\mu ,$$

where *M* is the degree of freedom of the mechanism, *n* is number of components including the rack, *g* is number of kinematic pairs, *f_i_* is the number of degrees of freedom for the *i*-th kinematic pair, and *μ* is the total number of over-constraints in the mechanism.

To analyze the number of motion pairs and components of the 3-RCU parallel mechanism, the number of components *n* is 8, the number of motion pairs *g* is 9, and the total number of over-constraints *μ* in the mechanism is zero. The sum of the degrees of freedom of the nine motion pairs is 15. Substitute these parameters into Equation (1), and the degree of freedom of the platform is 3.

## 3. The Design of Branch Chain 

### 3.1. Branch Chain Structure Design

**Figure 3:**
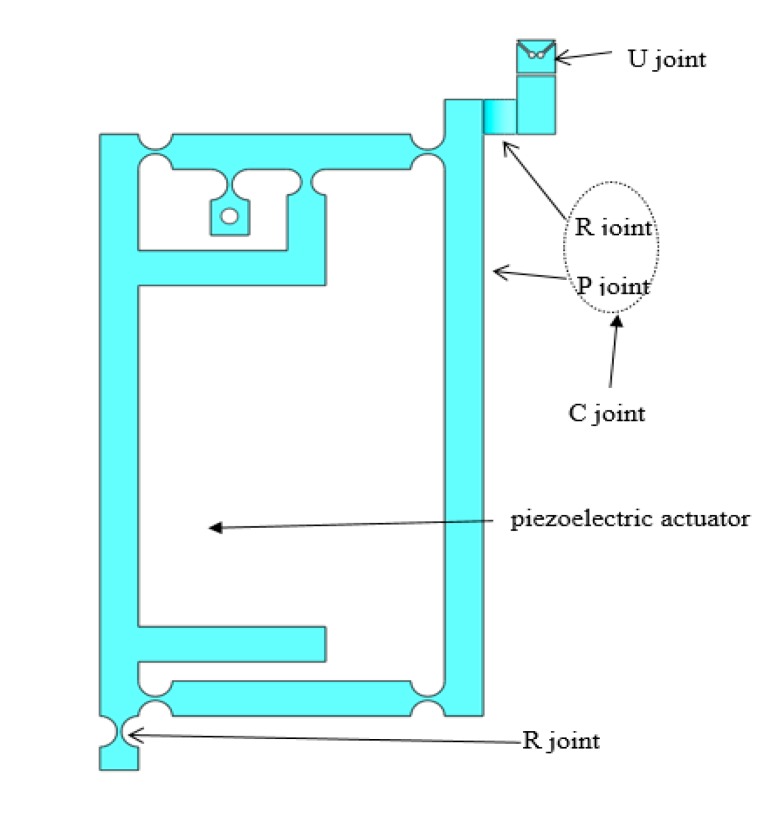
Single branch chain.

The single branch chain of the 3-RCU platform is shown in Figure 3. The motion pairs in the branches are flexible hinges instead of the traditional rigid motion pairs because the flexible hinges have small elastic deformation, higher displacement resolution, and fast speed response, which can avoid the free motion and mechanical friction of the platform during the movement.

The single branch chain is structurally composed of a flexible hook hinge U, a flexible cylindrical pair C including a micro-displacement amplification mechanism, and a flexible revolute pair R. The flexible hook hinge U can realize the rotation of two orthogonal axes, which can be realized by two flexible rotating pairs in series. Generally, the flexible hook hinge has two types: axis intersection and axis stagger. They have good compactness and high precision. This paper adopts the former with a simpler structure. 

Since the traditional rigid motion pair can realize the functions of movement and rotation, the flexible cylindrical pair C can be formed by a combination of a flexible moving pair and a rotating pair. The flexible moving pair adopts parallel four-bar flexible moving pair. Its guiding precision is high, its flexibility is concentrated, its movement condition is good, but its movement stroke is small [23]. When the piezoelectric actuator works, the displacement of the branch chain can be increased by the amplification mechanism, and the working space of the micro-operation platform is enlarged.

The PZTs (Model P-820.20) manufactured by PI Company of Germany (PI Company, Karlsruhe, Germany), which has a diameter of 9 mm and length of 44 mm. The driver can magnify and transfer its output displacement subtly. For the design of the PZTs, the size parameters of the piezoelectric actuators must be considered first. Therefore, the size of the branch chain must be designed reasonably, leaving enough space. Since PZT cannot withstand pressure, shear, torque, etc., a hemispherical sphere with a radius of 1.5 mm is designed on top of the driver to contact with the rod, which can ensure the safety of PZT and extend the service life [10].

**Figure 4:**
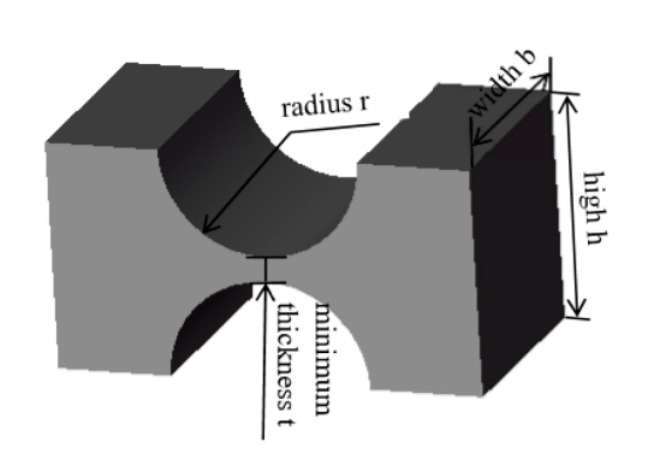
Straight round flexure hinge.

The flexure hinge of 3-RCU are all straight round flexure hinge. Figure 4 shows the structure of straight circular flexure hinge. It has high flexibility and a good rotation angle, which can better reduce the energy lost during the operation of the amplifying mechanism, increases the amplification ratio of the lever amplifying mechanism, and improves the overall output performance of the platform [10]. In addition, research of Hu, J.F. [24] on the performance of four kinds of flexure hinges and research of Wang, C.T. [25] on the stiffness characteristics of three kinds of flexible four-bar mechanisms can prove that the straight circular flexure hinge and the flexible four-bar mechanism with four straight circular flexure hinges have better comprehensive performance. In comparison with other hinges, the straight circular flexure hinge has the smallest shape variable, the highest precision, and the least stress. So, the amplifier and rotating pairs adopt a straight circular flexure hinge. The design parameters of the straight round flexible hinges are shown in Table 1. In Table 1, the *t* is the minimum thickness of the flexure hinge, *r* is the radius of the flexure hinge, *b* is the width of the flexure hinge, and *h* is the thickness of the flexure hinge.

### 3.2. Design of Amplification Mechanism 

**Figure 5:**
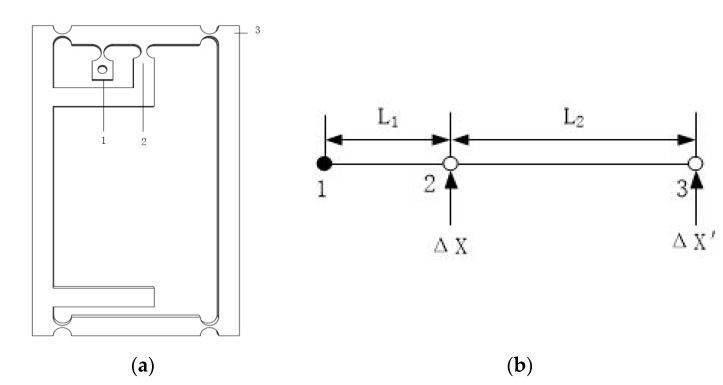
(**a**) Structure diagram of Amplification mechanism. (**b**) Schematic diagram of the lever amplification mechanism.

Scott–Russell amplification, lever amplification, and bridge displacement amplification are three flexible hinge amplification methods commonly used at this stage [12,26,27]. Because of the simple structure, good performance of the level magnification mechanism, and serious distortion of the magnification ratio of the multi-stage magnification mechanism [28], a new type of one-stage magnification mechanism with small space size is designed. Figure 5a is structural diagram of the lever amplifying mechanism, where 1, 2, and 3 respectively represent the fixed position, the input end, and the output end.

The lever amplifying mechanism is shown in Figure 5b. The solid point 1 is the fixed end of the amplifying mechanism, the distance between input end and the fixed end is *L*_1_, and the distance between the input end and the output end is *L*_2_. Δ*X* is the input end displacement and Δ*X*’ is the displacement produced by the output end. The theoretical amplification ratio of the mechanism is calculated by the principle of the lever amplification mechanism [29]:

(2) $$A=\frac{\Delta Χ}{\Delta {Χ}^{\prime }}=\frac{L_{1}+L_{2}}{L_{1}}$$

In the Equation (2), *L_1_* = 9mm, *L_2_* = 18.5 mm. We can get A ≈ 3.056.

## 4. Finite Element Analysis

In order to verify the reliability of the theoretical calculation results of the magnification ratio of the lever magnification mechanism and the rationality of the relevant performance of the micro-operation platform, the micro displacement magnification mechanism and the micro-operating platform need to be simulated and analyzed. Finite element analysis (FEA) is also known as the finite element method. From the perspective of mechanics, the complex continuum is divided into a finite number of elements in an imaginary way, and the finite number of elements are combined into an assembly in a certain way to replace the original continuum for research [30,31]. For the high-precision, complex, and nonlinear system of a flexible parallel robot, the finite element method takes into full account the elastic deformation of all part of the flexure hinge and can reflect the mechanical properties of the flexible parallel robot more correctly and comprehensively [32,33]. Create 3D models of magnifying mechanism and 3-RCU micro manipulation platform in SolidWorks (SolidWorks, Concord, MA, USA), save the file as parasolid (x.t.), then import the finite element simulation analysis software ANSYS Workbench (ANSYS, Pittsburgh, PA, USA) for simulation analysis.

The aluminium alloy (AL)7075-T6 (Alcoa, Pittsburgh, PA, USA) of aerospace aluminum alloy is used as the material for the micro-operation platform. The density of AL7075-T6 is 2810 kg/m^3^, the Poisson’s ratio is 0.33, the modulus of elasticity is 71,700 Pa and the yield strength of the material is 503 MPa.

The parameters of the P-820.20 piezoelectric actuators manufactured by the German PI company are as follows: the resolution is 0.3 nm, the maximum thrust is 50 N, the maximum stroke is 30 μm, the stiffness is 7 N/μm, and the no-load resonant frequency is 15,000 Hz.

### 4.1. Simulation Analysis of Amplification Mechanism

**Figure 6:**
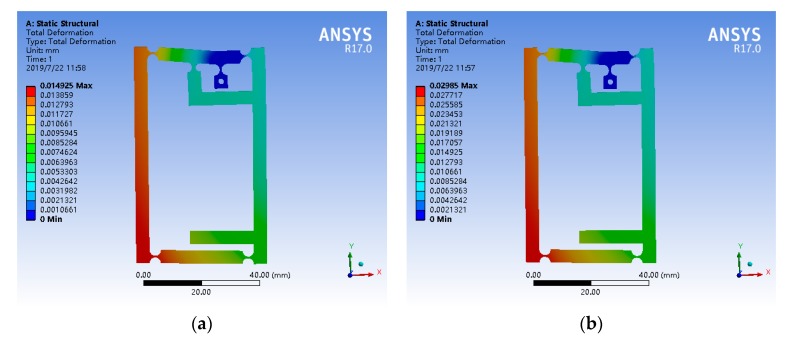
Simulation of amplifier mechanism. (**a**) Input displacement 5 μm. (**b**) Input displacement 10 μm.

The theoretical amplification ratio of the amplification mechanism has already known, and its reliability is verified by simulation analysis. Open the model in the ANSYS Workbench, add materials, set and modify material parameters, divide grids, apply constraints and loads, and then conduct finite element simulation analysis. The mesh size of the partition is set as 2 mm, and the fixed constraint is applied at the fixed hole of the fixed hinge. Loading 5 μm and 10 μm displacements at the input end of the amplification mechanism, respectively, the results of the static simulation of amplifier mechanism is shown in Figure 6. The output displacements from the simulation results were 14.925 μm and 29.850 μm, respectively. So, the simulation magnification of the mechanism is 2.985 and the margin of error is only 2.3%. The reason for this error is that the elastic deformation of the flexure hinge is not considered sufficiently, and the deformation of the flexure hinge and flexible bar are neglected in theoretical calculation.

### 4.2. Verification of Material Properties Selected for the Platform

**Figure 7:**
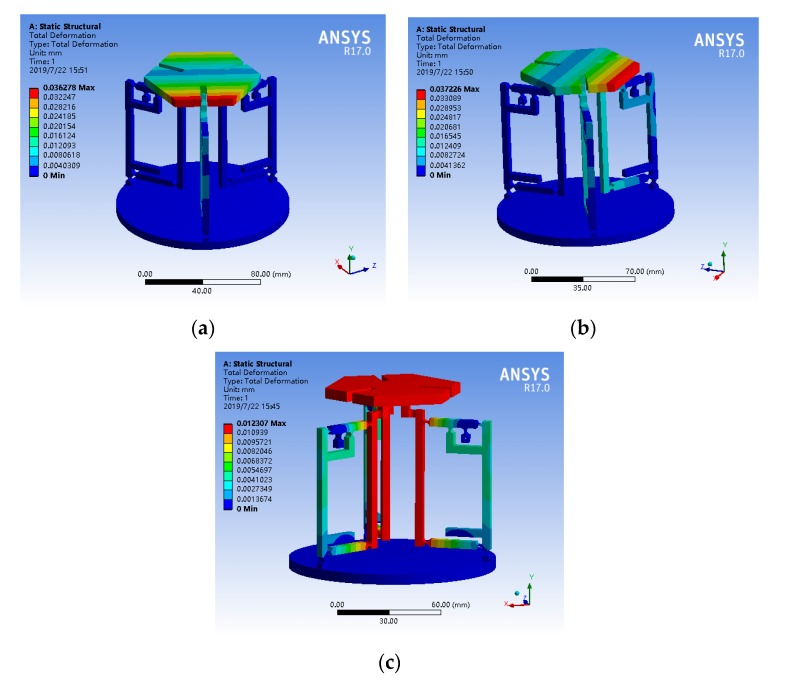
Simulation of amplifier mechanism. (**a**) One-branch was loaded displacement of 5 μm. (**b**) Two-branch were loaded with displacement of 5 μm. (**c**) Three-branch were loaded with displacement of 5 μm.

First of all, in the case of no load on the entire platform, the input ends of one branch chain, two branch chains, and three branch chains are respectively loaded with a displacement of 5 μm. The simulation results are shown in Figure 7. The maximum output displacements under the three loading modes were 36.278 μm, 37.226 μm, and 12.307 μm, respectively. Combined with the output displacement of the moving platform under three working conditions, it can be seen that each branch chain can work independently, and the platform has good motion decoupling. In the actual working process, loading different displacements and angles can make the micro-operating platform reach the ideal spatial position.

From the data of Figure 7c, the simulation magnification of the overall platform is 2.461. The reasons for this result are: the platform structure is more complicated, the error generated during simulation calculation is larger, there are more flexible hinges in the platform than the amplification mechanism, and more energy is absorbed by the flexible hinge.

**Figure 8:**
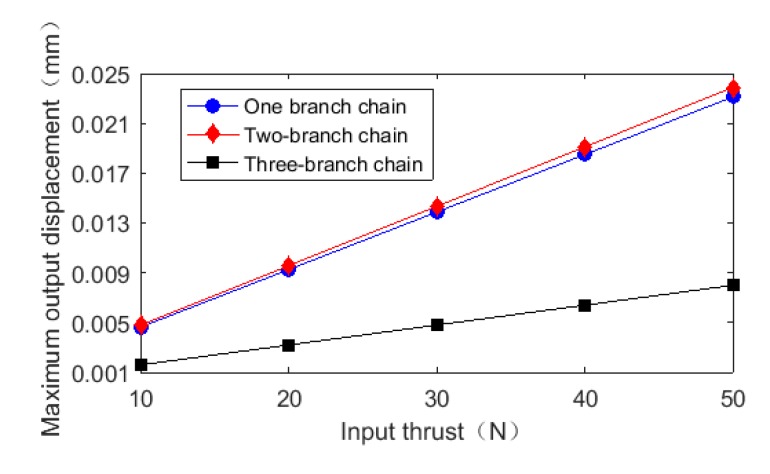
Relationship input thrust and maximum output displacement.

Then, the parameters of the selected piezoelectric actuator are verified by the stiffness of the whole mechanism under various working conditions. Determine whether the piezoelectric actuators can meet the demand of platform output characteristics, and verify the reliability of the platform. In the case of no load, thrust of 10 N, 20 N, 30 N, 40 N, and 50 N was loaded on the input end of one branch chain, two branch chains, and three branch chains of the platform, respectively, and the relationship between input thrust and output displacement is shown in Figure 8. In the case where one-branch chain, two-branch chain and three-branch chain are respectively applied with thrust, the rigidity of the mechanism is 2162.86 N/mm, 2093.01 N/mm, 6254.29 N/mm, respectively. The results of simulation are all lower than that of the actuator with a stiffness of 7000 N/mm (7 N/μm), indicating that the platform can overcome the stiffness of the external mechanism and generate thrust. In addition, the flexibility of the platform determines the carrying capacity of the platform and affects the positioning accuracy of the platform. That is, the higher the stiffness, the higher the positioning accuracy [34].

**Figure 9:**
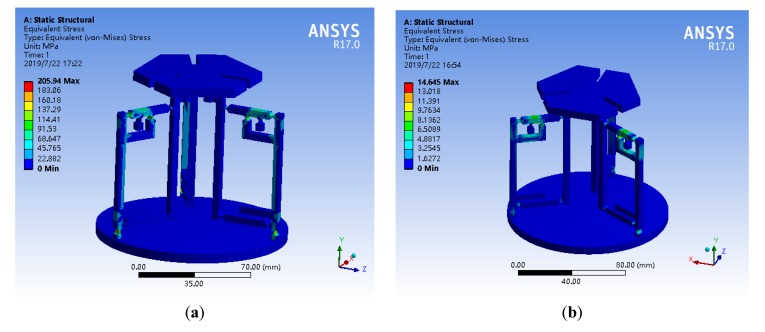
Static simulation of maximum stress value. (**a**) Three branches chain loaded displacement of 30 μm; (**b**) Three branches chain loaded thrust of 50 N.

Considering the micro-operating platform under extreme conditions, whether the maximum stress is within the yield strength range of the selected AL7075-T6 aerospace aluminum alloy, verify the safety of the platform work. The three branched chains were loaded with a maximum displacement of 30 μm and a maximum thrust of 50 N respectively. The static simulation of maximum stress value is shown in Figure 9. The simulation results show that the maximum stress of the former simulation is 205.94 MPa, and the maximum stress of the latter simulation is 14.645 MPa. The maximum stress values occurred at a circular flexure hinge and less than the yield strength of AL7075-T6 aviation aluminum alloy 503 MPa. Therefore, the micro-operation platform will not break during operation, which ensures the safety of the platform and the stability of the mechanism.

### 4.3. Modal Analysis

Finally, modal analysis is performed on the 3-RCU platform. The natural frequency is very important for the micro operation platform. The higher the natural frequency, the stronger the anti-interference ability of the micro operation platform, and the faster the dynamic response. Table 2 shows the first six natural frequencies of the mechanism obtained by modal analysis. The piezoelectric actuators P820.20 used in the platform has a natural frequency of 15,000 Hz, which is much larger than the natural frequency obtained by simulation. Therefore, the platform has better anti-interference ability. The platform will not resonate with PZTs driver during operation, which ensures the service life and safety of the platform.

## 5. Conclusions

This paper presents the design of a new 3-RCU flexible parallel micro operation platform. Carrying out theoretical calculations and finite element simulation analysis on the magnification of the lever amplification mechanism, the margin of error is just 2.3%. The decoupling of the platform under three different displacement conditions and the stiffness of the platform under three different conditions were simulated and analyzed. The maximum stress under the limit condition is obtained by loading the maximum displacement and the maximum thrust on the platform. Modal analysis was performed on the micro-operation platform to obtain the first six natural frequencies of the mechanism. A series of simulation results show that the design of the platform is reasonable and reliable, and the platform has large stroke, high accuracy, good safety, and good dynamic performance. For the magnification mechanism, the design of a better performing magnification mechanism still needs in-depth research. The parameters of the platform and flexure hinge are not optimized in this paper, and will be worth studying in the future. In addition, considering whether the flexible parallel micro-operation platform can be better applied to manufacturing and whether the relevant design of the platform is of great significance to the development of the discipline, it is also necessary to solve the workspace, conduct kinematics analysis, dynamics analysis, and error analysis, which will be the core of the next work.

## Figures and Tables

**Table 1:** Design parameters of straight round flexure hinge.

Parameter	*t*	*r*	*b*	*h*
Value (mm)	0.5	2.0	4.5	4.5

**Table 2:** The first six natural frequencies.

No.	Natural Frequencies (Hz)	No.	Natural Frequencies (Hz)
1	129.59	4	518.96
2	176.34	5	525.30
3	177.17	6	551.60
